# Congenital heart disease missense mutations in the TBX5 DNA-binding domain alter thermal stability and DNA-binding affinity

**DOI:** 10.1093/g3journal/jkaf174

**Published:** 2025-08-02

**Authors:** Alejandro Rivera-Madera, Edwin G Peña-Martínez, Jean L Messon-Bird, Diego A Pomales-Matos, Oswaldo L Echevarría-Bonilla, Leandro Sanabria-Alberto, Esther A Peterson-Peguero, José A Rodríguez-Martínez

**Affiliations:** Department of Biology, University of Puerto Rico, Río Piedras Campus, San Juan, PR 00931, United States; Department of Biology, University of Puerto Rico Cayey, Cayey, PR 00736, United States; Department of Biology, University of Puerto Rico, Río Piedras Campus, San Juan, PR 00931, United States; Department of Biology, University of Puerto Rico, Río Piedras Campus, San Juan, PR 00931, United States; Department of Biology, University of Puerto Rico, Río Piedras Campus, San Juan, PR 00931, United States; Department of Biology, University of Puerto Rico, Río Piedras Campus, San Juan, PR 00931, United States; Department of Biology, University of Puerto Rico, Río Piedras Campus, San Juan, PR 00931, United States; Department of Biology, University of Puerto Rico, Río Piedras Campus, San Juan, PR 00931, United States; Department of Biology, University of Puerto Rico, Río Piedras Campus, San Juan, PR 00931, United States

**Keywords:** transcription factor, missense mutations, TBX5, thermal stability, DNA-binding affinity

## Abstract

Missense mutations can alter the biochemical properties of proteins, including stability, structure, and function, potentially contributing to the development of multiple human diseases. Mutations in *TBX5*, a transcription factor necessary for heart development, are among the causes of congenital heart diseases. However, further research on biophysical and biochemical mechanisms is needed to understand how missense mutations in transcription factors alter their function in regulating gene expression. In this work, we applied in vitro and in silico approaches to understand how 5 missense mutations in the TBX5 T-box DNA-binding domain (I54T, M74V, I101F, R113K, and R237W) impact protein structure, thermal stability, and DNA-binding affinity to known TBX5 cognate binding sites. Differential scanning fluorimetry showed that mutants I54T and M74V had decreased thermal stability, mutants I101F and R113K had increased stability, and R237W had no significant effect on stability. Additionally, DNA-binding affinity decreased for all 5 missense mutants when evaluated in vitro for known TBX5 genomic binding sites within regulatory elements of *Nppa* and *Camta1* genes. Structural modeling of the TBX5 predicted altered protein conformations and stability due to the loss or gain of amino acid residue interactions. Together, our findings provide biophysical and biochemical mechanisms that can be further explored to establish causality between TBX5 missense mutations and the development of congenital heart diseases.

## Graphical Abstract

**Graphical Abstract:**
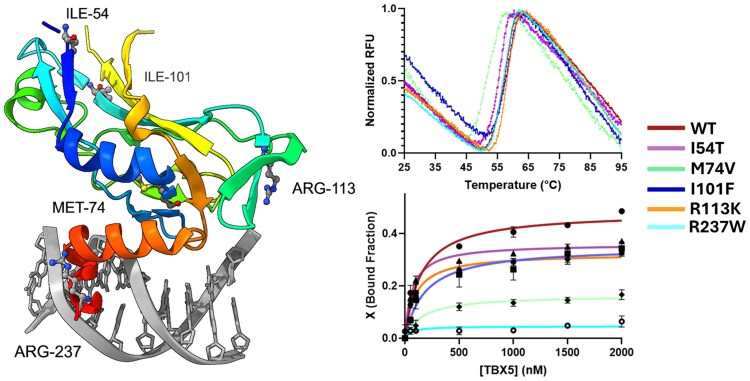

## Introduction

Missense mutations, genetic variants that cause a change in an amino acid, can adversely affect protein structure and function, leading to pathogenic outcomes (Stefl et al. 2013; Selvaraj and Piramanayagam 2019; Stein et al. 2019). Missense mutations have been linked to numerous human genetic diseases, such as sickle cell anemia and cystic fibrosis (Pauling et al. 1949; Ingram 1958; Rommens et al. 1989). Missense mutations have been shown to alter biochemical mechanisms, such as protein stability, protein folding, protein activity, intermolecular interactions, posttranslational modifications, and cellular localization (Petrosino et al. 2021; Sirp et al. 2021; Xiong et al. 2022; Pan et al. 2024; Serghini et al. 2024). At a cellular level, mechanisms like gene expression have been shown to be altered by missense mutations of regulatory proteins, like transcription factors (TFs) (Freeman et al. 1994; Brandt et al. 2012; Campagne et al. 2012; Käppel et al. 2021).

TFs are sequence-specific DNA-binding proteins that regulate the spatial and temporal gene expression. Previous work linking TF mutants to human diseases has reported altered structure, thermal stability, and function of multiple TFs (Bullock et al. 1997; Kirk et al. 2007; Campagne et al. 2012; Chen et al. 2019; Dai et al. 2020; Khadiullina et al. 2022). For over 30 years, oncogenic missense mutations have been known to alter the stability of p53 (Lane and Crawford 1979; Bullock et al. 1997; Khadiullina et al. 2022). In an in vivo system, Kirk et al. (2007) used *Xenopus laevis* (African clawed frog) embryos to link clinical TBX20 missense mutations to disruption of developmental processes, such as gastrulation. Although further insight is required to understand how missense mutations can alter TF function and regulatory activity, understanding changes in TF stability and DNA binding is a step forward in establishing causal mechanisms of many human diseases.

TBX5, also known as T-box TF 5, is an evolutionarily conserved TF involved in heart and limb development (Fan et al. 2003; Bruneau 2008; Zaidi and Brueckner 2017). The *TBX5* gene is expressed during embryonic development in multiple layers (epicardium, myocardium, and endocardium) and chambers (atrial appendages and left ventricle) of the developing heart (Chapman et al. 1996; Steimle and Moskowitz 2017). TBX5 binds to the consensus sequence 5′-AGGTGT-3′ and regulates multiple cardiac genes, such as the *natriuretic peptide precursor A* (*Nppa*) and *calmodulin-binding transcription activator* (*Camta1*), to regulate gene expression and promote cell differentiation into cardiac tissue and cardiac function (Habets et al. 2002; Mori et al. 2006; Song et al. 2006). Knockouts of *TBX5* in mice have shown decreased expression of cardiac genes needed for heart development (e.g. *Nppa* and *CX40*) and even developmental arrest or embryo lethality (Bruneau et al. 2001; Davidson and Erwin 2006; Warren et al. 2011). Missense mutations of TBX5 have been associated with multiple types of congenital heart diseases (CHDs), such as Holt–Oram syndrome (HOS), atrial septal defect (ASD), and ventricular septal defect (VSD) (Beddington et al. 1992; Horb and Thomsen 1999; Ghosh 2001; Papaioannou 2001; Dreßen et al. 2016). Missense mutations can alter TBX5 function by impairing molecular interactions with known binding partners (e.g. NKX2-5, GATA4, Mef2C, and SRF) active during heart development (Vincentz et al. 2008; Maitra et al. 2009; Bruneau 2013). Additionally, mutations in the TBX5 DNA-binding domain (DBD) can alter TF-DNA interactions with *cis*-regulatory elements (CRE, e.g. promoters and enhancers) and disrupt the expression of genes crucial for heart development (Freeman et al. 1994; Ang et al. 2016; Käppel et al. 2021). For example, one of the first reported TBX5 mutants, R237W, showed decreased binding to the human *ANF* promoter (Basson et al. 1999; Fan et al. 2003). TBX5 mutants have been previously reported to have altered DNA-binding affinity for known binding sites (Ghosh 2001; Fan et al. 2003; Baban et al. 2014; Dreßen et al. 2016; Khatami et al. 2018; Møller Nielsen et al. 2024).

**Fig. 1.**
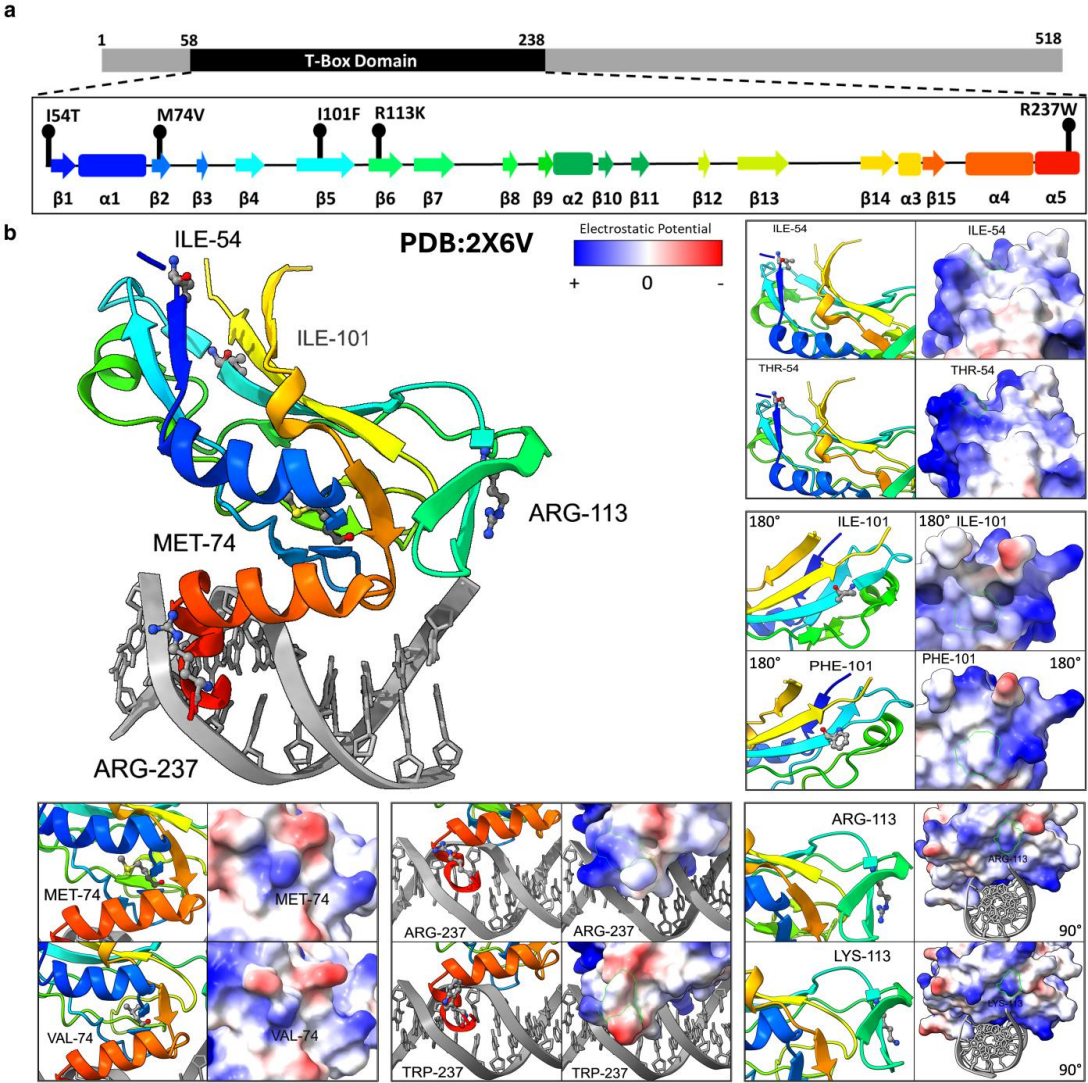
Diagram of TBX5 missense mutations evaluated in this work. a) Location of mutations within the TBX5 T-box domain. Alpha helices and beta sheets within the T-box domain are represented with rectangles and arrows, respectively. Colors correspond to the position in the crystallography structure. b) Amino acid substitution in each mutant in cartoon model and surface model showing electrostatic potential. Mutant residues modeled with MutationExplorer (Phillipp et al. 2024) using PDB ID:2X6V.

In this work, we evaluated the thermal stability and DNA-binding activity of 5 CHD-associated missense mutants in the TBX5 T-box DBD (I54T, M74V, I101F, R113K, and R237W; Fig. 1a). All 5 CHD-associated missense mutants in the T-box domain of TBX5 were identified from the ClinVar (M74V) (Landrum et al. 2014) and OMIM (Hamosh et al. 2005) databases, and most were identified in patients or family trios with HOS (I54T and R237W) (Basson et al. 1999; Yang et al. 2000), ASD (I101F), and VSD (R113K). Recombinant TBX5 T-box missense mutants were expressed and purified through affinity chromatography. Functional evaluation of TBX5 missense mutants was performed through differential scanning fluorimetry (DSF) and electrophoretic mobility shift assay (EMSA). We observed changes in TBX5 mutant melting temperature (T_m_) and binding affinity for known TBX5 genomic binding sites. All 5 variants were computationally modeled to predict structure, amino acid residue interactions, and thermodynamic stability changes (Fig. 1b). Our results provide the first evidence of altered thermal stability for mutants I54T, I101F, R113K, and DNA-binding affinity for mutants M74V, I101F, and R113K. Furthermore, our work closely follows the approach by Stirnimann et al. (2010), including R237W in our experiments as a control for altered thermal stability and DNA-binding affinity, as was previously described (Fan et al. 2003; Stirnimann et al. 2010). This suggests a role in congenital malformations for CHD-associated missense mutations in TBX5 by disrupting its regulatory function.

## Materials and methods

### Cloning, expression, and purification of TBX5 T-box domain

The TBX5 T-box domain plus 10 amino acids flanking at each end (Leu48-Ser248) was cloned into the pET-51b(+) bacterial overexpression vector using Gibson Assembly (New England Biolabs, #E5510S). pET51b(+) adds an N-terminal Strep-Tag-II and C-terminal 10X His-Tag. Five missense mutations were generated using the Quick-change II Site-Directed Mutagenesis kit (Agilent Technologies, #200523). Sequences and primers are available in Supplementary Tables 1 and 2. All plasmids were verified through long-read whole-plasmid sequencing (Plasmidsaurus Inc.) and transformed into BL21 DE3 *E. coli* (New England Biolabs #C2530H or Millipore Sigma #70956-4). First, 50 mL Luria broth (Sigma-Aldrich, #L3022) cultures were grown for 16 h at 37 °C, shaking at 230 rpm, followed by inoculation of 500 mL of Terrific broth (Sigma-Aldrich, #T0918) with 10 mL of the overnight culture and grown at 37 °C. Once the culture reached OD_600_ between 0.5 and 0.8, protein expression was induced by adding 1 mM IPTG and culturing at 18 °C while shaking. After 20 h, the culture was centrifuged at 2800× *g* for 5 min at 4 °C, and the pellet was frozen at −80 °C overnight.

The pellet was resuspended with 40 mL of column buffer, and 4 mL (10% of volume) of 5 M NaCl was added. The sample was then sonicated for four cycles at 40% amplitude for 30 s (QSONICA, part no. Q125), centrifuged (2800 × *g*, 30 min, 4 °C), and the supernatant was incubated with 2 mL of Ni-NTA resin (Qiagen, # 30210) (1 h, 4 °C, orbital shaking). The column was equilibrated with 20 mL of column buffer (500 mM NaCl, 20 mM Tris-HCl, pH 8.0, 0.2% Tween-20, 30 mM imidazole, and EDTA-free protease inhibitor) (Thermo Scientific # A32965). The supernatant was then passed through the column twice, followed by 3 washes with increasing concentrations of imidazole (30 mM, 50 mM, and 100 mM) in column buffer. The protein was eluted 6 times with 1.8 mL of elution buffer (500 mM NaCl, 20 mM Tris-HCl, pH 8.0, 0.2% Tween-20, 500 mM imidazole). Buffer exchange to binding buffer (50 mM NaCl, 10 mM Tris-HCl, pH 8.0, and 10% glycerol) was performed using Amicon Ultra Centrifugal Filter, 3 kDa concentrators (Millipore Sigma, #UFC5003).

Finally, the purification was evaluated with SDS-PAGE and western blot using Mini-PROTEAN TGX Precast Protein Gels (Bio-Rad, #4561086) as seen in Supplementary Fig. 1. Samples were prepared with 4× Laemmli Sample Buffer (Bio-Rad #1610747) containing β-mercaptoethanol for a total volume of 20 μL (5 μL 4× loading buffer + 15 μL sample), heated at 95 °C for 5 min, and resolved for 1 h at 120 V at room temperature. The gel was stained using Coomassie Brilliant Blue R stain (Sigma-Aldrich, #B0149). For the western blot analysis, contents from the SDS-PAGE gel were transferred to a PVDF membrane using the Bio-Rad Turbo Transfer System protocol in a Trans-Blot Turbo for 3 min at 25 V. Membranes were blocked using 5% milk in 1× TBST buffer for 1 h in orbital shaking and incubated overnight with 1:10,000 dilution of horse radish peroxidase-conjugated anti-His mouse monoclonal antibody (Novus Biologicals, AD1.1.10). The SDS-PAGE and western blot analysis results were imaged using Azure Sapphire Biomolecular Imager (Azure Biosystems).

### DFS

DSF (Jeremy Johnson et al. 2014) was used to evaluate the thermal stability of the TBX5 T-box domain and its missense mutants with the Applied Biosystems QuantStudio 3 Real-Time PCR System, 96-well, 0.2 mL (#A28137), using MicroAmp Optical 8-Tube strips (Applied Biosystems #4316567 and #432302). Samples were heated from 25 to 95 °C with a ramp of 0.05 °C/s and measured with a 470 ± 15 nm/520 ± 15 nm excitation/emission filter. Protein stocks of 10 µM in binding buffer (50 mM NaCl, 10 mM Tris-HCl, pH 8.0, and 10% glycerol) were diluted to 5 µM in a 20 µL reaction with 5× SYPRO Orange Dye (Invitrogen S6650), and the volume was completed with binding buffer. Melting curve raw data from the DSF melting assays were exported from the equipment and analyzed using DSFworld (Wu et al. 2024). Normalized raw data were plotted against the temperature (°C) using GraphPad Prism 10 to obtain melting curves for each wild-type–mutant pair. T_m_ values and graphs represent the average of 6 to 9 replicates per protein. Changes in melting point (ΔT_m_) were obtained by subtracting the wild-type T_m_ (Supplementary Fig. 3) from the mutant T_m_. In addition, statistical significance was determined through an unpaired parametric *t*-test of each mutant compared to the wild type. Specific reagents used are listed in Table 1.

**Table 1.** Reagents table.

Reagent or resource	Source	Identifier
Software and bioinformatic tools		
PRISM 10.2.2	GraphPad	N/A
DynaMut2	https://biosig.lab.uq.edu.au/dynamut2/	doi: 10.1002/pro.3942
RaSP	https://github.com/KULL-Centre/_2022_ML-ddG-Blaabjerg/	doi: 10.7554/eLife.82593
PremPS	https://lilab.jysw.suda.edu.cn/research/PremPS/	doi: 10.1371/journal.pcbi.1008543
iMutant 2.0	https://folding.biofold.org/i-mutant/i-mutant2.0.html	doi: 10.1093/nar/gki375
mCSM	https://biosig.lab.uq.edu.au/mcsm/stability	doi:10.1093/bioinformatics/btt691
Missense3D	https://missense3d.bc.ic.ac.uk/missense3d/	doi: 10.1016/j.jmb.2023.168374
MutationExplorer	https://proteinformatics.uni-leipzig.de/mutation_explorer/	doi:10.1093/nar/gkae301
AlphaFold3	https://alphafoldserver.com/welcome	doi:10.1038/s41586-024-07487-w
TM-Align	https://zhanggroup.org/TM–align/	doi: 10.1093/nar/gki524
Recombinant DNA		
pET-51b(+)	Millipore Sigma	71553
Oligonucleotides	Integrated DNA Technologies	See Supplementary Table 2
IRDye 700 fluorophore		
Bacterial strains		
BL21 DE3 *E. coli*	New England Biolabs	C2530H
	Millipore Sigma	70956-4
Chemical and other reagents		
Luria broth	Sigma-Aldrich	L3022
Terrific broth	Sigma-Aldrich	T0918
β-d-1-Thiogalactopyranoside (IPTG)	Sigma-Aldrich	I6758
Ni-NTA resin	Qiagen	30210
Sodium chloride (NaCl)	Fisher Scientific	S671-3
UltraPure 1 M Tris-HCI, pH 8.0	Invitrogen	15568025
Tween-20	Sigma-Aldrich	P9416
Imidazole	Sigma-Aldrich	I2399
Pierce EDTA-free protease inhibitor	Thermo Scientific	A32965
Amicon Ultra Centrifugal Filter, 3 kDa MWCO	Millipore Sigma	UFC500396
Mini-PROTEAN TGX Precast Protein Gels	Bio-Rad	4561086
4× Laemmli Sample Buffer	Bio-Rad	1610747
β-Mercaptoethanol (BME)	Millipore Sigma	444203
Coomassie Brilliant Blue R stain	Sigma-Aldrich	B0149
Anti-His mouse monoclonal antibody	Novus Biologicals	AD1.1.10
Glycerol	Sigma-Aldrich	G5516
10× TBE buffer (Tris/boric acid/EDTA)	Bio-Rad	1610770
ProtoGel (30%)	National Diagnostics	EC890
Ammonium persulfate	Sigma-Aldrich	A3678
N,N,N′,N′-Tetramethylethane-1,2-diamine (TEMED)	Thermo Scientific	17919
MicroAmp Optical 8-Tube strips	Applied Biosystems	4316567/432302
SYPRO Orange protein gel stain	Invitrogen	S6650
Commercial assays		
Gibson Assembly	New England Biolabs	E5510S
Quick-change II Site-Directed Mutagenesis kit	Agilent Technologies	200523

### EMSAs

DNA-binding activity was assessed through EMSA with 2 known binding sites for TBX5. Assays were performed with 40 bp sequences derived from the *Nppa* and the *Camta1* promoters, with an additional 20 bp constant region to add IRDye 700 fluorophore (Integrated DNA Technologies) through a primer extension reaction (available in Supplementary Table 2). Binding reactions of 20 µL in binding buffer (50 mM NaCl, 10 mM Tris-HCl, pH 8.0, and 10% glycerol) were prepared with 7 protein concentration points (0, 50, 100, 500, 1000, 1500, and 2000 nM). Acrylamide gels went through a prerun at 85 V for 15 min before loading samples at 35 V. Samples were incubated for 30 min at 30 °C and 30 min at room temperature before resolution in a 6% native polyacrylamide gel for 1.5 h at 75 V in 0.5× TBE (89 mM Tris HCl, 89 mM boric acid, 2 mM EDTA, pH 8.4). The results were imaged using the Azure Sapphire Biomolecular Imager with an excitation/emission of 658 nm/710 nm (Azure Biosystems) and analyzed as previously described (Peña-Martínez, et al. 2025). Fold change in the bound fraction was calculated by dividing the maximal binding fraction (B_max_) value of the wild-type TBX5 T-box domain by the B_max_ value of the corresponding mutant. Specific reagents used are listed in Table 1.

### In silico prediction of missense mutant's thermodynamical stability and structure

Predictions of changes in thermodynamic stability were performed for the missense mutants compared to the wild-type TBX5 using web-based programs that calculate changes in Gibson's free energy (ΔΔG) and/or protein structure. We predicted thermodynamic stability using DynaMut2 (Rodrigues et al. 2021), RaSP (Blaabjerg et al. 2023), PremPS (Chen et al. 2020), iMutant 2.0 (Capriotti et al. 2005), and mCSM (Pires et al. 2014) (Table 3). Residue substitution structure models were generated with MutationExplorer (Fig. 1b). In addition, we predicted the structure of DNA-bound full-length TBX5 and its DBD mutants, similar to the wild type in PDB ID:2X6V in the Google DeepMind AlphaFold3 Server (https://alphafoldserver.com/welcome) (Abramson et al. 2024), and established alignments between wild type and each mutant for the structure ranked first using TM-Align (https://zhanggroup.org/TM–align/; Fig. 4a) (Zhang and Skolnick 2005). This tool helped to evaluate the similarity of the folding position of each residue by calculating the TM-Score, which varies in a range from 0 to 1, where 1 indicates a perfect similarity between the compared structures. Changes in amino acid interactions and structural damage predictions were performed with DynaMut2 (Rodrigues et al. 2021) and Missense3D (Ittisoponpisan et al. 2019), respectively (Fig. 4b and Table 3) (Philipp et al. 2024). All predictions were performed using chain A from PDB 2X6V, which represents the protein in bound conformation to the DNA. Pathogenicity likelihood was predicted using MutPred2 (Pejaver et al. 2020) using the 2X6V TBX5 structure (Supplementary Table 3). AlphaFold 3 structure predictions were generated using the sequence from Uniprot ID: Q99593 including residues 1 to 518 for the full-length protein and 53 to 238 for the T-box domain.

## Results

### Missense mutations in TBX5 T-box domain alter thermal stability

**Fig. 2.**
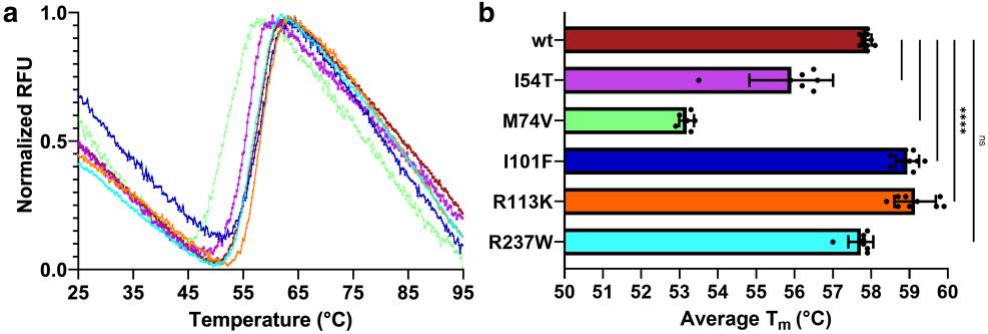
Evaluation of mutant TBX5 T-box domain thermal stability by differential scanning fluorimetry. a) Melting curves were generated for the wild-type T-box domain and each missense mutant. Melting curves normalization and T_m_ analysis were performed using DSFworld. RFU: relative fluorescent units. b) T_m_ quantification and statistical analysis through unpaired *t*-test (**** = *P* < 0.0001). Color scheme: wild-type T-box domain (red), I54T (purple), M74V (green), I101F (blue), R113K (orange), and R237W (cyan).

We evaluated the in vitro thermal stability of the wild-type TBX5 T-box domain and 5 missense mutants (I54T, M74V, I101F, R113K, and R237W) with DSF. In DSF, protein thermal stability is determined by measuring the fluorescence of SYPRO Orange during protein thermal denaturation. Changes in thermal stability were determined by comparing the melting temperatures (T_m_) between wild-type TBX5 and mutants. Four of the evaluated mutants had statistically significant changes in melting temperatures (T_m_) (Fig. 2). Wild-type TBX5 T-box domain has a T_m_ of 57.9 ± 0.2. Mutants I54T and M74V showed a decrease in thermal stability with T_m_ values of 55.9 °C ± 1.1 (ΔT_m_ = −2.0 °C) and 53.2 °C ± 0.2 (ΔT_m_ = −4.7 °C), respectively. In contrast, mutants I101F and R113K showed an increase in thermal stability with T_m_ values of 58.9 °C ± 0.3 (ΔT_m_ = 1.0 °C) and 59.1 °C ± 0.5 (ΔT_m_ = 1.2 °C), respectively. No significant changes in thermal stability were observed for R237W, as evaluated through a *t*-test (Fig. 2b). Changes in thermal stability for each mutant are summarized in Table 2.

**Table 2.** Summary of changes in thermal stability and DNA-binding affinity validated in vitro.

Protein	T_m_ (°C)	ΔT_m_ (°C)	Significance (*t*-test)	Binding fold change	
				ANF	camta
WT	57.9 ± 0. 1	N/A	N/A	N/A	N/A
I54T	55.9 ± 1.1	−2.0	**** (*P* < 0.0001)	−1.65	−1.34
M74V	53.2 ± 0.2	−4.7	**** (*P* < 0.0001)	−3.27	−2.93
I101F	58.9 ± 0.3	1.0	**** (*P* < 0.0001)	−1.26	−1.38
R113K	59.1 ± 0.5	1.2	**** (*P* < 0.0001)	−2.87	−1.49
R237W	57.7 ± 0.3	−0.2	ns (*P* = 0.2274)	−3.76	−10.52

### TBX5 missense mutants decrease binding affinity for known binding sites

To further determine the biochemical implications of these missense mutations, we analyzed changes in binding affinity for known TBX5 genomic binding sites. TBX5 T-box domain DNA-binding affinity was evaluated in vitro through EMSA using fluorescently labeled oligonucleotides containing 40 bp derived from the *Nppa* and *Camta1* promoter. Binding curves were constructed using 7 protein concentrations (0, 50, 100, 500, 1000, 1500, and 2000 nM) and averaging 3 experimental replicates.

**Fig. 3.**
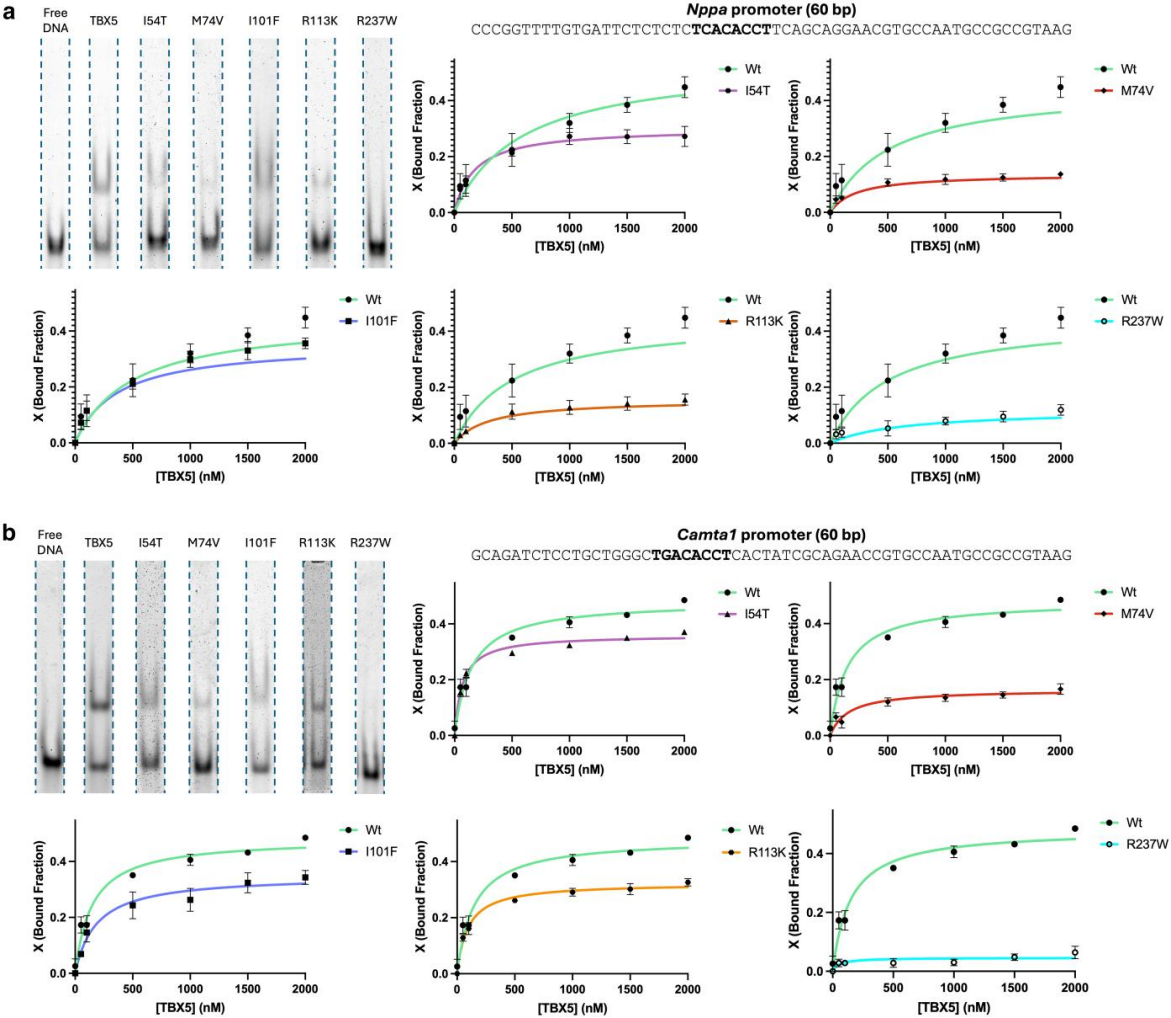
Evaluation of in vitro DNA-binding affinity on TBX5 missense mutants for known binding sites. Binding assays for a) mouse *Nppa* and b) mouse *Camta1* promoters. The figure shows EMSA gel shift bands at 2000 nM (upper left) and binding curves for each T-box mutant. Gel images were derived from Supplementary Fig. 4. Color scheme: wild-type T-box domain (red), I54T (purple), M74V (green), I101F (blue), R113K (orange), and R237W (cyan). Sequences used for fluorescent probes are at the top of each figure with the wild-type TBX5 binding motif in bold.

All 5 missense mutants showed a decrease in DNA-binding affinity to both the *Nppa* (Fig. 3a) and the *Camta1* promoters (Fig. 3b) compared to the wild-type TBX5 T-box domain. Mutant R237W had the greatest impact on DNA-binding, with a 3.76- and 10.52-fold change for known binding sites within the *Nppa* and *Camta1* promoters, respectively. This was followed by M74V with a 3.27- and 2.93-fold change decrease for *Nppa* and *Camta1*. Mutant I54T had a 1.65- and 1.34-fold decrease for *Nppa* and *Camta1*, respectively. Mutant R113K had a 2.87- and 1.49-fold change decrease for *Nppa* and *Camta1*. Finally, mutant I101F had the lowest impact on DNA binding with a 1.26- and 1.38-fold change decrease for *Nppa* and *Camta1*, respectively. Fold changes in binding for each mutant are summarized in Table 2.

### TBX5 missense mutations are predicted to decrease thermodynamic stability and alter structural features

We performed predictions of changes in thermodynamic stability (ΔΔG), structural damage, changes in amino acid interactions, and 3D structure using tools with web user interfaces (Table 3). All mutations were predicted to decrease the thermodynamic stability relative to the wild-type TBX5 T-box domain. Mutant M74V was the only mutation predicted to have structural damage, with the loss of a buried hydrogen bond donated from Lys226 and a cavity alteration, as predicted with Missense3D. Additionally, all TBX5 missense mutants were predicted to be pathogenic through the gain or loss of biochemical features (e.g. loss of posttranslational modifications and tertiary structures) and protein function (e.g. DNA binding, metal binding, and allosteric/catalytic sites) (Supplementary Table 3). Mutant M74V had the highest pathogenicity score (0.92/1.00), with predicted altered DNA/metal binding and PTMs sites (e.g. methylation site in lysine 78). Conversely, I54T was the least pathogenic (0.72/1.00), which predicted altered metal binding and stability. Among the remaining mutants, I101F (0.79/1.00) had a predicted loss of acetylation and methylation at lysine 99 and altered metal binding capacity. Mutants R113K (0.80/1.00) and R237W (0.87/1.00) had new acetylation and methylation sites and altered DNA-binding activity. Our in vitro findings support the altered stability predicted for I54T and DNA binding for M74V, R113K, and R237W. Protein interactions are mediated by contacts with specific amino acid residues, such as TF binding to DNA or enzymatic recognition for posttranslational modifications. As expected, protein function and biochemical properties can change by altering specific amino acid interactions with other molecules.

**Table 3.** In silico prediction on structural and thermodynamic stability changes for TBX5 mutants.

	Structural change				Thermodynamical stability change (ΔΔG)				
Protein	Missense3D	TM-Score (0,1]		DynaMut2 (Interactions)	RaSP	PremPS	iMutant 2.0 (37 °C, pH = 8.0)	mCSM	DynaMut2
		T-Box domain	Full-Length Protein						
I54T	No structural damage detected	0.99	0.45	−Val56 (HPh)	2.55 ↓	0.98 ↓	−2.05 ↓	−1.877 ↓	−1.39 ↓
M74V	Structural damage detected Cavity altered Buried H-bond breakage	0.98	0.53	−Thr72 (HPh), −Phe84 (H, VDW, P), −Trp64 (HPh), −Lys226 (H)	1.01 ↓	1.64 ↓	−1.37 ↓	−1.426 ↓	−1.80 ↓
I101F	No structural damage detected	0.99	0.47	−Val137 (HPh), −Leu103 (HPh)	0.84 ↓	0.73 ↓	−0.66 ↓	−1.743 ↓	−1.66 ↓
R113K	No structural damage detected	0.99	0.50	+Met176 (HPh), +Lys126 (H), +Val123 (H)	0.71 ↓	0.87 ↓	−1.63 ↓	−1.160 ↓	−1.41 ↓
R237W	No structural damage detected	0.99	0.53	−Glu228 (H), −Asn230 (P)	0.34 ↓	0.12 ↓	−0.01 ↓	−0.804 ↓	−0.88 ↓

Gained amino acid interactions are marked with a plus (+), while lost interactions are marked with a minus (−). Specific amino acid interactions are described as follows: hydrophobic (HPh), hydrogen (H), van der Waals (VdW), and polar (P). Mutants with a predicted decrease in thermodynamic stability are marked with a downward arrow (↓) next to the computational score.

**Fig. 4.**
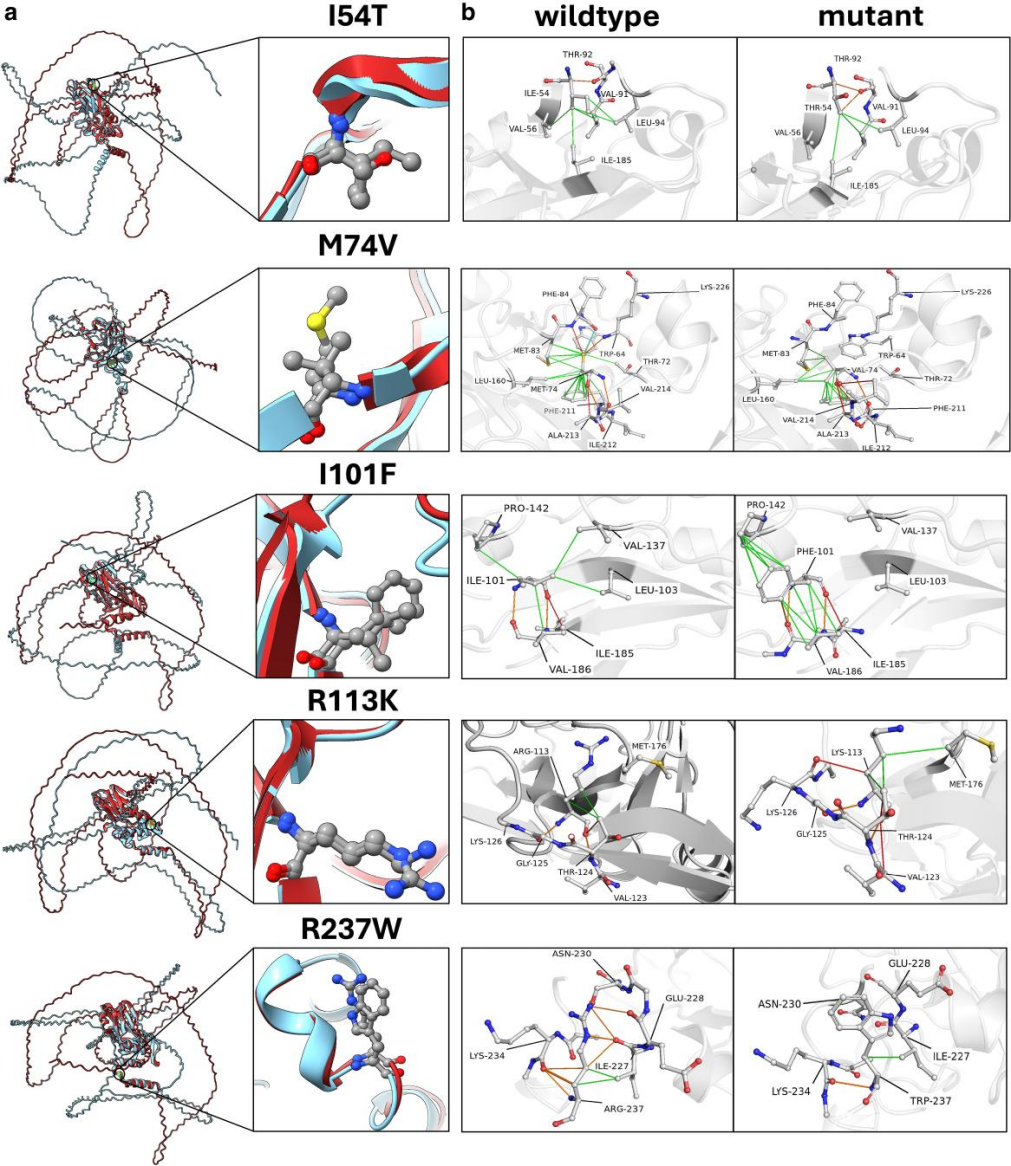
In silico modeling of amino acid interactions in the TBX5 T-box domain. a) Structural similarities between the wild-type TBX5 and missense mutants. Structures were predicted using Alphafold3 and aligned with TM-Align: wild-type TBX5 (red) and mutant TBX5 (blue). b) Interactions between amino acids of wild-type T-box domain (left) with its mutant counterpart (right). Images are zoomed in and centered on the amino acid substitution. Specific interactions are illustrated as follows: hydrophobic interactions (green), hydrogen bonds (red), polar bonds (orange), ionic bonds (yellow), and Van der Waals (light blue). Modeled using PDB ID:2X6V (Stirnimann et al. 2010) in DynaMut2 (Rodrigues et al. 2021).

Additionally, we predicted DNA-bound structures of wild-type and mutant full-length TBX5 and T-box domain using Alphafold 3 and compared them using Tm-Align (Fig. 4a; Supplementary Fig. 2). TM-Score values (0,1] vary in a range of 0.45 to 0.53, which indicates a low similarity in the folded structure between the predicted wild-type and mutant full-length structures (Table 2). In contrast, predicted structures for the wild-type T-box domain and mutants show higher similarity in their fold with TM-Score values ranging from 0.98 to 0.99. To further understand the in silico predictions, amino acid interactions in the TBX5 T-box domain were visualized using DynaMut2 and compared to all 5 missense mutants (Fig. 4b). DynaMut2 is a machine learning model that predicts the effects of point mutations on protein stability and dynamics through protein folding and structure analysis. All mutants resulted in at least one broken or new interaction between neighboring amino acid residues. When compared to the wild-type TBX5 T-box domain, mutant I54T lost a hydrophobic interaction with V56. Mutant M74V lost 2 hydrophobic interactions with residues T72 and W64 and 3 interactions with residue F84 (hydrogen bond, van Der Waals, and polar). Mutant I101F lost 2 hydrophobic interactions with residues V137 and L103. Mutant R113K was the only one predicted to create interactions with new residues by forming 2 new hydrogen bonds with residues K126 and V123 and a hydrophobic interaction with M176. While conserving the residue interaction, mutant I101F increased the number of hydrophobic bonds formed with P142 and V186. Mutant R237W lost a hydrogen bond with residue E228 and a polar interaction with residue N230.

## Discussion

Rare coding variants in genes that are essential for heart development, such as *TBX5*, *NKX2-5*, and *GATA4*, contribute to the etiology of CHDs (Dallapiccola et al. 1994; Zaidi and Brueckner 2017; Khatami et al. 2018; Morton et al. 2022; Xiao et al. 2024). Hence, understanding the molecular mechanisms of mutations in proteins needed for proper heart development, such as cardiac TFs, is key to understanding CHD etiology. In this work, we aimed to understand how the missense mutants of the cardiac TF TBX5 can alter its thermodynamic stability, DNA-binding properties, and structural properties. Toward this goal, we focus our work on 5 TBX5 missense mutations (I54T, M74V, I101F, R113K, and R237W) in the DBD present in CHD patients from ClinVaR (Landrum et al. 2014) (Fig. 1a and b). Toward this goal, we applied a series of computational tools to perform in silico predictions on structural changes [46,47] and thermodynamic stability (Capriotti et al. 2005; Pires et al. 2014; Chen et al. 2020; Rodrigues et al. 2021; Blaabjerg et al. 2023) for all 5 TBX5 missense mutants and validated them in vitro (Tables 2 and 3).

First, we evaluated how these predicted structural changes may alter TBX5 stability and function, and we cloned, expressed, and purified wild-type and mutant TBX5 T-box domains (Supplementary Fig. 1). We performed thermal stability assays on the wild-type T-box domain and all 5 missense mutants to evaluate changes in their T_m_ (Fig. 2). Two missense mutations (I54T and M74V) decreased thermal stability, while 2 (I101F and R113K) resulted in an increase after in vitro evaluation through DSF. M74V had the greatest decrease in thermal stability (ΔT_m_ = −4.7 °C), which was expected since it is a large hydrophobic residue buried in the hydrophobic core and there are 5 predicted disrupted interactions, leading to structural damage as predicted by Missense3D. Conversely, R113K had the highest gain in thermal stability (ΔT_m_ = 1.2 °C) while being the only mutant with novel predicted residue interactions. In these cases, with opposite outcomes, M74V and R113K had a respective loss and gain of hydrophobic interactions and hydrogen bonds, which greatly contribute to protein folding and structural stability, respectively (Gromiha and Selvaraj 2004; Stefl et al. 2013; Newberry and Raines 2019). Similarly, mutants I54T and R237W resulted in a decrease in thermal stability (ΔT_m_ = −2.0 and −0.2 °C, respectively), while I101F increased (ΔT_m_ = 1.0 °C). Overall, 3 of the thermodynamic stability predictions were consistent (I54T, M74V, and R237W), which decreased just as all the computational tools predicted. The findings and data available from this work (available in Supplementary File 1) can be used to train or improve existing models to cover some of the current limitations in predicting protein stability at a single amino acid resolution.

To further understand how missense mutations may alter TBX5 function, we next evaluated changes in DNA-binding affinity to known TBX5 genomic binding sites. Through in vitro binding assays, we observed a decrease in affinity to known TBX5 binding sites within the *Nppa* and *Camta1* promoters, 2 genes involved in heart development (Fig. 3; Supplementary Fig. 4). Mutant M74V had the greatest decrease in DNA-binding affinity, with a 3.27- and 2.93-fold decrease for *Nppa* and *Camta1*, respectively. This decrease may be derived from the significant impact on M74V thermal stability as well as the predicted structural damage, limiting the formation of interactions needed for TF-DNA binding. However, the greatest decrease in binding affinity for both *Nppa* and *Camta1* probes came from mutant R237W. Substitution of the positively charged arginine residue to the nonpolar bulky tryptophan can hinder the formation of ionic interactions with the negative charges of the DNA phosphate backbone (Sousa et al. 2010; Solovyev et al. 2015; Tomaž et al. 2022). Similarly, mutant R113K also had an arginine residue substitution and considerably decreased affinity for both the *Nppa* and *Camta1* sequences. Although the substitution was for a lysine, another positively charged residue, previous work has shown that arginine-rich domains are important for the binding of some TFs (Shailesh et al. 2018; Lorton and Shechter 2019; Brodsky et al. 2021; Wu et al. 2021). Mutants I54T and I101F decreased the DNA-binding affinity of the T-Box domain while showing decreased and increased thermal stabilities, respectively. Protein-DNA binding requires conformational flexibility, which can be impaired by increased rigidity (Tokuriki and Tawfik 2009; Dodonova et al. 2020; Pan et al. 2010; Weiler et al. 1998). In the case of mutants I101F and R113K, we hypothesize that their increase in thermal stability impaired their flexibility and reduced conformational dynamics needed for protein-DNA binding. This tendency was previously observed in work involving the vnd/NK-2 homeodomain where mutations at residue 54 increase the thermal stability of the protein by ≥2 °C while reducing the DNA-binding affinity of the TF. Our findings are consistent with previous work done in TBX5 missense mutants (reduced thermal stability of M74V and DNA-binding for I54T and R237W) and can complement past research on protein–protein interactions, gene expression, and cellular localization (Basson et al. 1999; Yang et al. 2000; Fan et al. 2003; Stirnimann et al. 2010). Through this approach, we have demonstrated that missense mutations within the T-Box domain of TBX5 can impact TF-DNA binding affinity. Not only can mutant TFs lose affinity for known binding sites, but they also open the possibility of having novel binding sites in the human genome and dysregulating genes outside their regulatory network (Freeman et al. 1994; Barrera et al. 2016; Aditham et al. 2021).

AlphaFold modeling and subsequent alignment demonstrate a decrease in folding similarity between the wild-type and mutant structures when the analysis is performed with the entire protein sequence versus the T-box domain alone (Table 3). TM-Scores obtained for the full-length structure predictions (0.47 to 0.53) are still above the threshold of random similarity detection by TM-Align (0.30) and may be low due to the large intrinsically disordered regions of TBX5. The two terminal regions have IDR domains that are predicted to be different for each mutant prediction. These observations highlight the importance of evaluating the impact of missense mutations in the full-length protein and not only in defined motifs, especially considering intrinsically disordered regions in involved in the identification of TFs binding sites (Jonas et al. 2025 ).

All 5 missense mutant structures are predicted to have changes in amino acid residue interactions within the T-box domain (Fig. 4). Four mutants (I54T, M74V, I101F, and R237W) lost residue interactions important for protein secondary structures formation and maintenance (e.g. hydrogen bonds and hydrophobic interactions), whereas one mutant (R113K) gained interactions. From our computational modeling of TBX5 mutants, we observed changes in the electrostatic potential of the mutant DBDs, which can hinder interactions with the negatively charged phosphate backbone of DNA molecules. Additionally, computational predictions of biochemical features predicted altered stability (I54T), metal/DNA binding (I54T, M74V, I101F, R113K, and R237W), structural features/folding (M74V, I101F, R113K, and R237W), and posttranslational modification (M74V, I101F, R113K, and R237W). Specifically, mutations were predicted to alter PTMs by gaining or losing methylation and acetylation marks, which can hinder TF transcriptional activity, stability, and protein–protein interactions. As such, all 5 missense mutations were predicted as likely pathogenic mutants (Supplementary Table 3). Furthermore, previous work has highlighted the biochemical and genetic mechanisms underlying the etiology of TBX5 mutant disease. For example, I54T and R237W have been previously shown to alter subcellular localization and NKX2-5 interactions (Fan et al. 2003). TBX5 interacts with other cardiac TFs in a cooperative manner to regulate cardiac gene expression (Hiroi et al. 2001; Maitra et al. 2009). Disruptions of these TF-TF interactions, potentially caused by TBX5 mutants, could dysregulate genes crucial for heart development, such as ANF, which has been shown to decrease expression with mutant cardiac TFs (Fan et al. 2003; Goetze et al. 2020; Audain et al. 2021).

To summarize, we evaluated the impact of TBX5 missense mutations on protein structure, thermal stability, and DNA-binding affinity through a combined in silico and in vitro approach. Computational T-box domain structure modeling revealed insights into amino acid residue interactions with potential implications for protein folding and stability. Through thermal stability and DNA-binding assays, we observed changes in the TBX5 T-box domain properties crucial to maintaining biological function. Together, our findings provide biophysical and biochemical mechanisms that can be further explored to establish causality between TBX5 missense mutations and the development of CHDs. We found no direct correlation between altered thermal stability and DNA-binding activity. Only 3 out of 5 mutants (I54T, M74V, and R237W) showed proportional changes between thermal stability and binding. Furthermore, we provide novel findings on thermal stability (I54T, I101F, R113K, and R237W) and DNA binding (M74V and R113K). Our comprehensive approach is scalable to other mutations in TFs crucial for organ development, furthering research toward diagnosis and treatments for multiple types of birth defects.

## Supplementary Material

jkaf174_Supplementary_Data

## Data Availability

All data are available in the published manuscript and its online Supplementary File 1. This file includes the raw and normalized DSF data, melting temperature values for both datasets, and results for in silico analysis performed with MutPred2. Supplemental material available at G3 online.
