# Supplementary material for: Congenital heart disease missense mutations in the TBX5 DNA-binding domain alter thermal stability and DNA-binding affinity
Source: G3 (Bethesda). 2025 Aug 2;15(10):jkaf174. doi: 10.1093/g3journal/jkaf174 (PMC12588321; doi:10.1093/g3journal/jkaf174)
Supplement: jkaf174_Supplementary_Data [file jkaf174_supplementary_data.zip › Supplementary_Figures_and_Tables_G3-2025-405947.docx]

**Supplementary Materials**
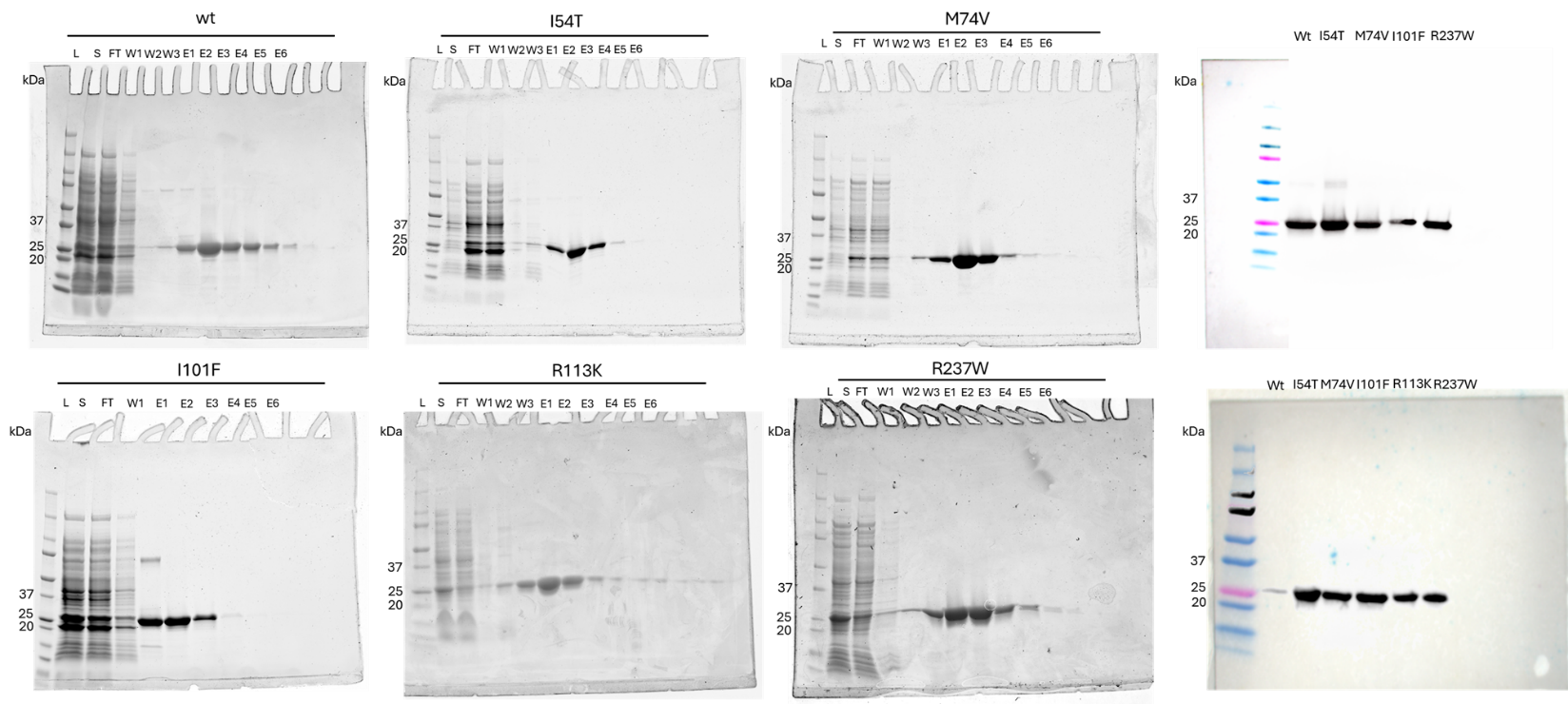


**Supplementary Figure 1:** T-box domain and mutant expression, purification, and western blot. Ladder (L), Supernatant (S), Flowthrough (FT), Wash (W), Elutes (E).

**
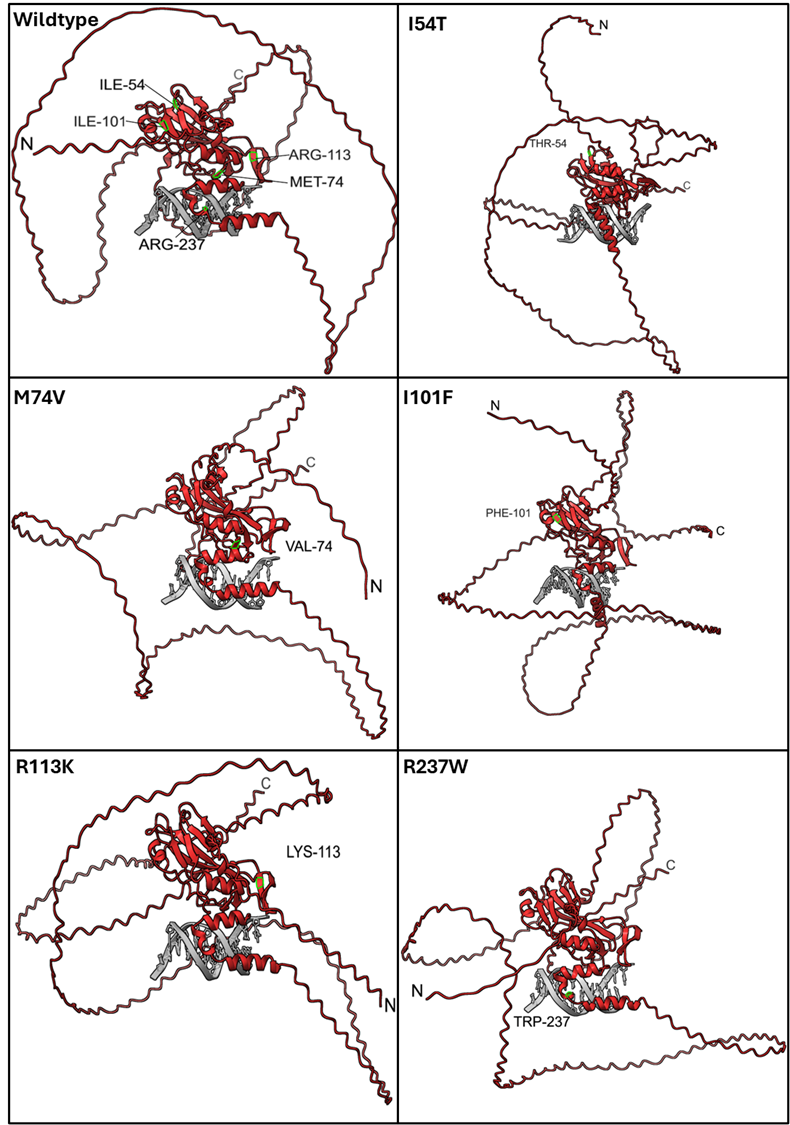
Supplementary Figure 2:** Full-length TBX5 wildtype and mutant structural predictions performed with Alphafold3. Evaluated and mutated residues are outlined in green. The DNA sequence used is the same as PDB ID: 2X6V.


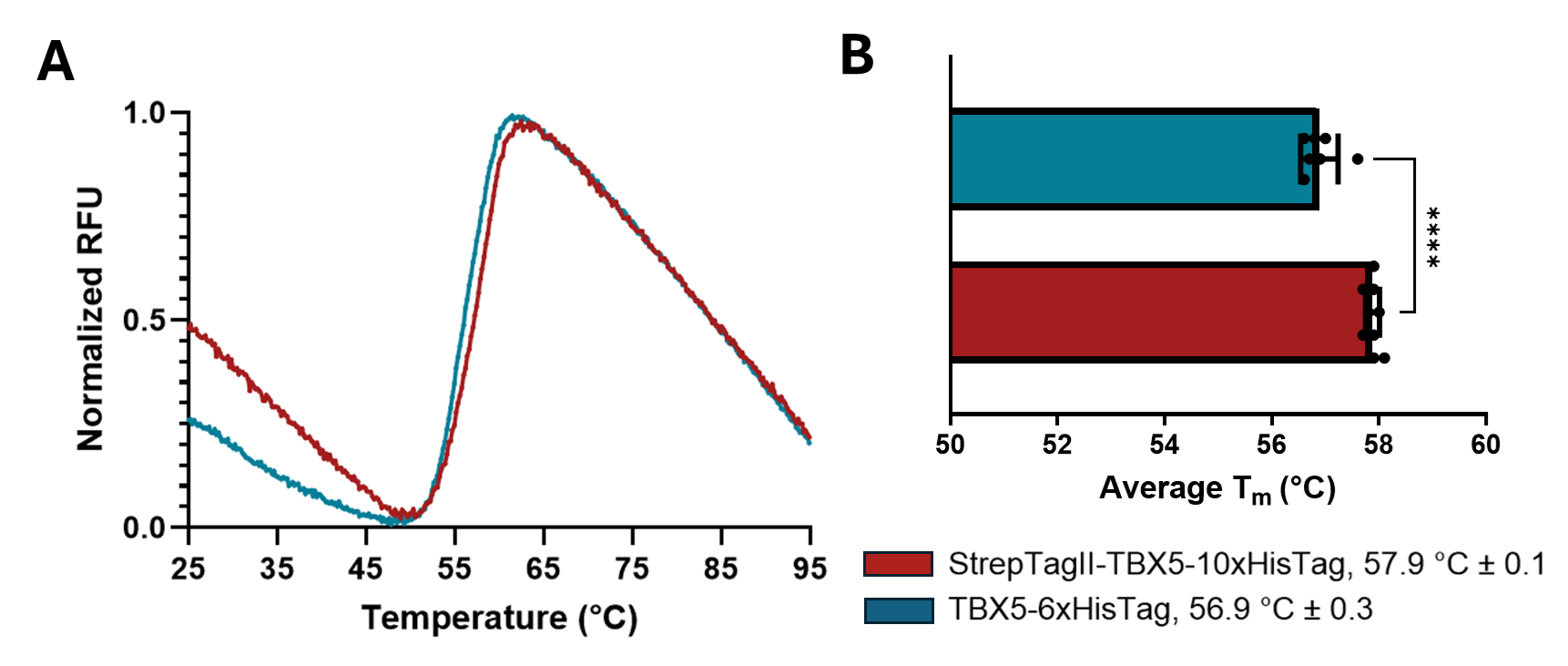


**Supplementary Figure 3**: Wildtype T-box domain melting curves created through DSF. Melting curve assays were optimized using wildtype T-box domains with different purification tags. Color scheme: wildtype T-box domain with N-terminal strep tag and C-terminal 10x His-tag used in this work (red), wildtype T-box domain with a 6x His-Tag (blue).

**
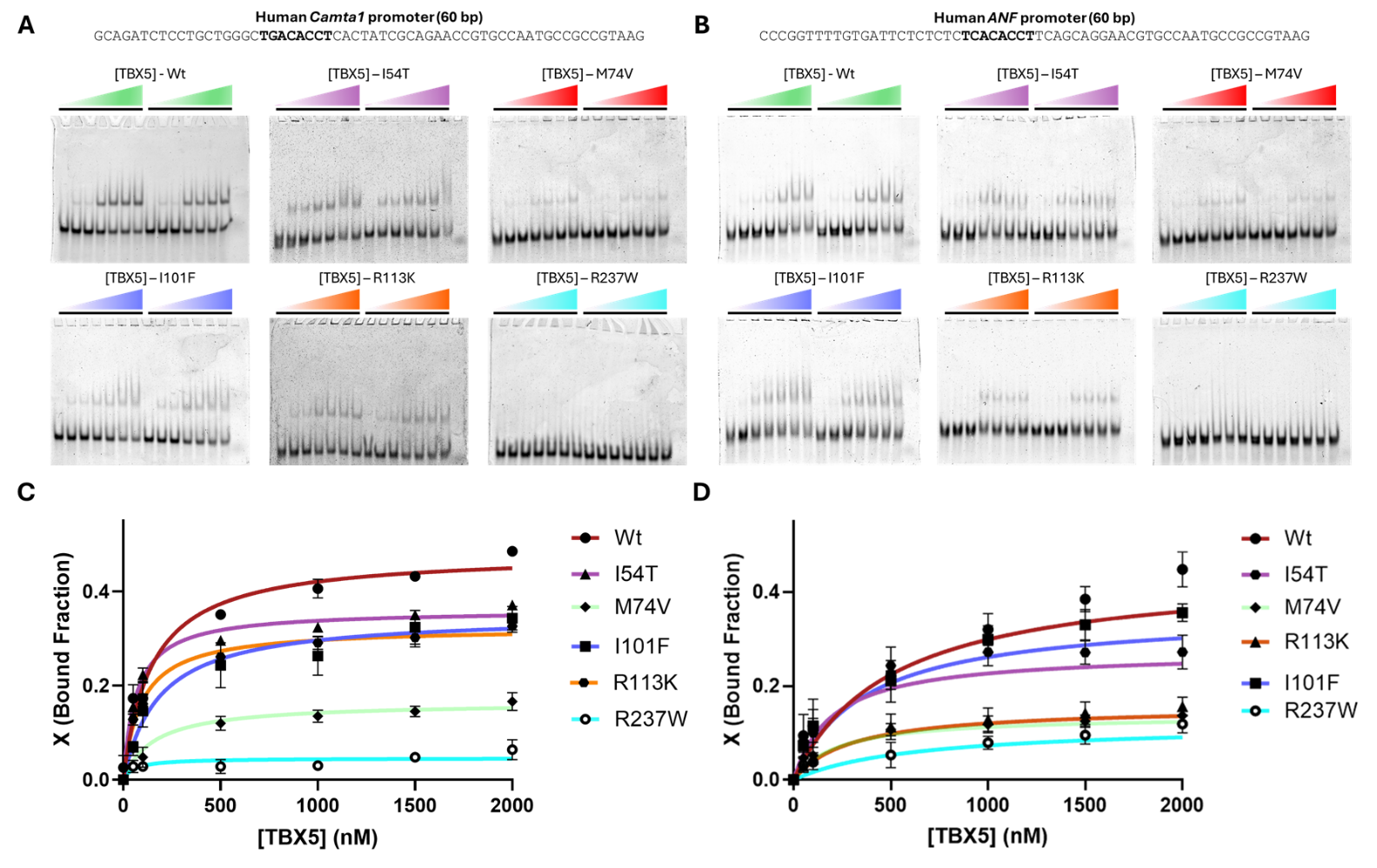
**

**Supplementary Figure 4:** Representative EMSA gels used in this work A representative EMSA for wildtype T-box domain and mutants to evaluate binding to **A**) *Nppa* and **B**) *Camta1* to generate binding curves for **Figure 4**. Merged binding curves for **C**) *Nppa* and **D**) *Camta1* were generated to compare binding for all five missense mutants. Color scheme: Wildtype T-box domain (green), I54T (purple), M74V (red), I101F (dark blue), R113K (orange), and R237W (light blue/cyan). Sequences used for fluorescent probes are at the top of each figure with the wildtype TBX5 binding motif in bold.

**Supplementary Table 1:** Amino acid sequence for wildtype and mutant T-box domain used in this work. Missense mutations are marked as underline amino acids.

**
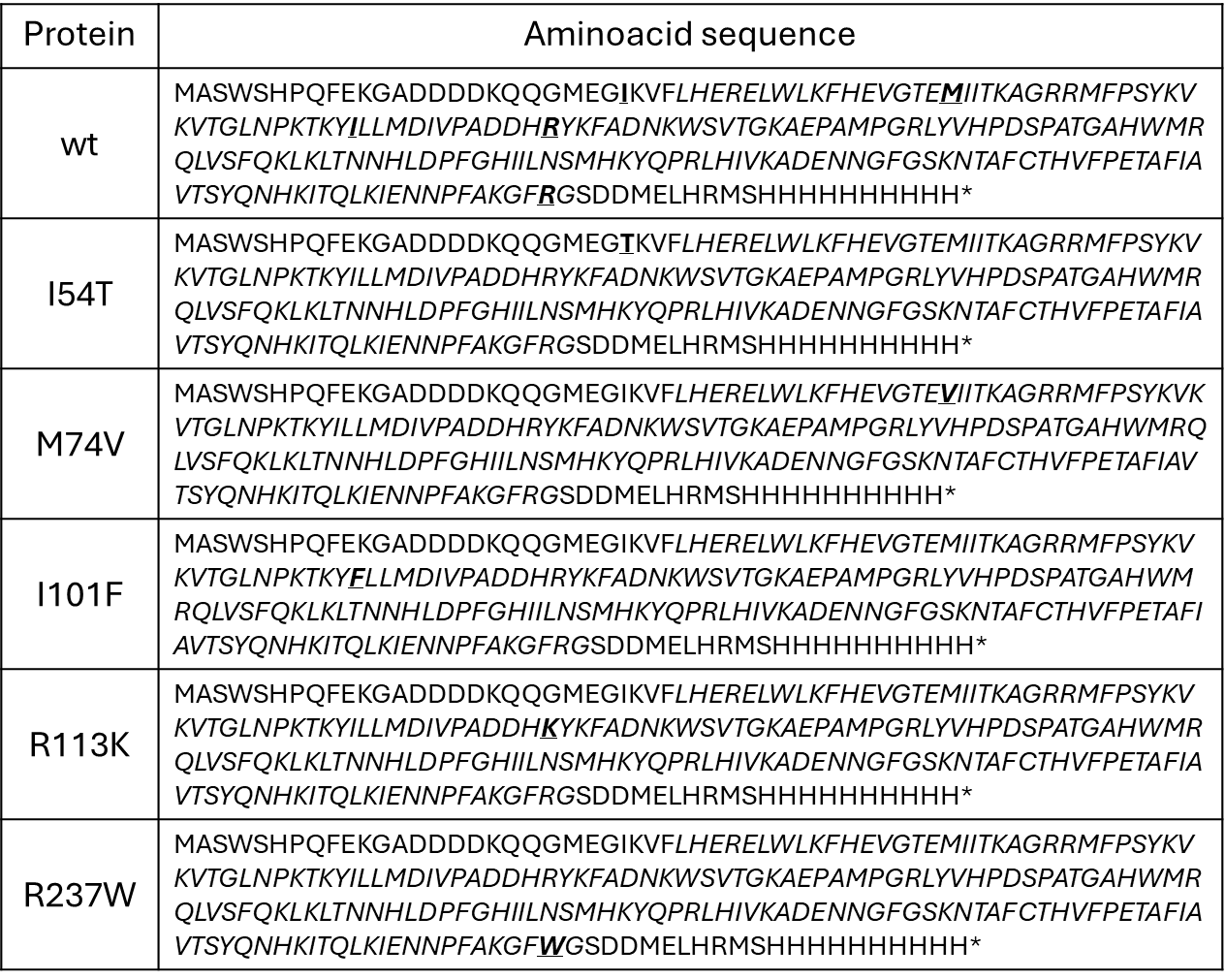
**

**Supplementary Table 2:** List of oligos used in this work.
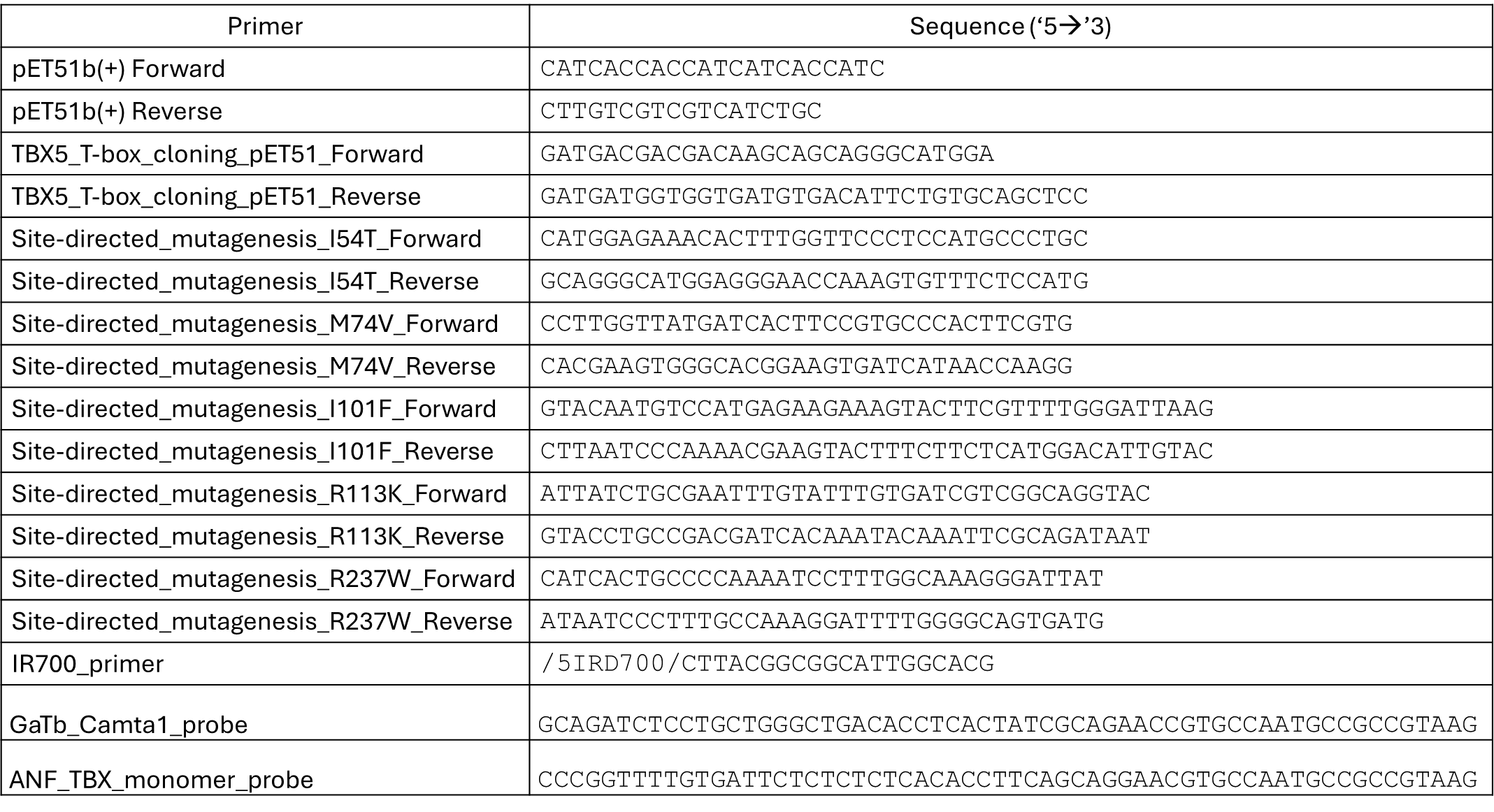


**Supplementary Table 3**: Pathogenicity predictions through MutPred2. Gain of function and features are highlighted as green, while losses are highlighted as red.

| **Mutation** | **MutPred2 Score (0,1)** | **Predicted pathogenic mechanism (p-value ≤ 0.05)** | | | |
| --- | --- | --- | --- | --- | --- |
|  |  | Mechanism | Probability | p-value |  |
| **I54T** | 0.715 | Altered Metal binding | 0.17 | 0.04 |  |
|  |  | Altered Stability | 0.11 | 0.04 |  |
| **M74V** | 0.924 | Gain of Allosteric site at T72 | 0.28 | 3.7e-03 |  |
|  |  | Altered Metal binding | 0.24 | 0.02 |  |
|  |  | Altered DNA binding | 0.22 | 0.02 |  |
|  |  | Gain of Methylation at K78 | 0.17 | 9.7e-03 |  |
|  |  | Loss of Catalytic site at E73 | 0.12 | 0.03 |  |
| **I101F** | 0.789 | Gain of Allosteric site at D105 | 0.29 | 2.2e-03 |  |
|  |  | Altered Metal binding | 0.26 | 0.02 |  |
|  |  | Loss of Relative solvent accessibility | 0.26 | 0.03 |  |
|  |  | Loss of Acetylation at K99 | 0.25 | 0.01 |  |
|  |  | Loss of Catalytic site at D105 | 0.11 | 0.03 |  |
|  |  | Loss of Methylation at K99 | 0.11 | 0.03 |  |
| **R113K** | 0.800 | Gain of Relative solvent accessibility | 0.29 | 0.01 |  |
|  |  | Altered Metal Binding | 0.27 | 7.6e-03 |  |
|  |  | Gain of Acetylation at R113 | 0.27 | 6.3e-03 |  |
|  |  | Altered Ordered interface | 0.24 | 0.04 |  |
|  |  | Altered DNA binding | 0.19 | 0.03 |  |
|  |  | Gain of Methylation at K115 | 0.10 | 0.04 |  |
| **R237W** | 0.874 | Loss of Intrinsic disorder | 0.41 | 0.02 |  |
|  |  | Altered DNA binding | 0.34 | 5.0e-04 |  |
|  |  | Gain of Allosteric site at R237 | 0.33 | 7.0e-04 |  |
|  |  | Altered Disordered interface | 0.28 | 0.03 |  |
|  |  | Loss of Helix | 0.28 | 0.03 |  |
|  |  | Loss of Acetylation at K234 | 0.28 | 5.6e-03 |  |
|  |  | Gain of Loop | 0.27 | 0.04 |  |
|  |  | Loss of Methylation at K234 | 0.18 | 9.4e-03 |  |
|  |  | Gain of Catalytic site at K234 | 0.13 | 0.03 |  |
